# Antimicrobial resistance and genetic diversity of *Salmonella enterica* from eggs

**DOI:** 10.1002/fsn3.1126

**Published:** 2019-08-01

**Authors:** Tengfei Xie, Gang Wu, Xujun He, Zengzhe Lai, Huatong Zhang, Jing Zhao

**Affiliations:** ^1^ Research Center of Plant Pest Management and Bioenvironmental Health Technology Guangdong Eco‐engineering Polytechnic Guangzhou China

**Keywords:** antibiotic resistance, food safety, molecular typing, *Salmonella*

## Abstract

*Salmonella enterica* is a common foodborne pathogen responsible for major global health problems such as paratyphoid fever and gastroenteritis. Here, we report the prevalence, antibiotic resistance phenotypes, serotypes, and molecular subtyping of *Salmonella* isolated from eggs in Guangdong, China. Out of 1,000 egg samples, 54 (5.40%) were positive. *S.* Enteritidis made up the largest proportion of samples with 11 serotypes. Antimicrobial susceptibility test indicated that most strains were resistant to β‐lactam, aminoglycoside, and tetracycline antibiotics (27.00%–40.00%). There were 37 STs based on MLST typing. MLST and ERIC‐PCR classified 54 isolates into three and five clusters, respectively, which revealed the genetic relatedness and diversity. In conclusion, frequent monitoring of eggs for *Salmonella*, antibiotic resistance profiles and genetic diversity is essential for improving food safety.

## 1 INTRODUCTION

*Salmonella enterica* is a major foodborne pathogen that infects approximately 9 million people worldwide and is responsible for 155,000 deaths annually, causing serious economic losses (Huang et al., 2016; Li, Ye, et al., 2017). It is well known that this pathogen is mainly spread by unsafe handling of uncooked animal food products including egg, chicken, and pork (Gillespie, O'Brien, Adak, Ward, & Smith, 2005; Wang et al., 2012). As an important foodborne pathogen in China, *Salmonella* was responsible for approximately 70%–80% of foodborne pathogenic outbreaks (Wang, Zheng, & Wang, 2007). Typhoidal *Salmonella* causes severe and life‐threatening diseases, while nontyphoidal *Salmonella* is associated with self‐limiting diseases such as gastroenteritis, but still severe systemic infections occur in infants, the elderly, and immune‐compromised individuals (Ceyssens, Mattheus, Vanhoof, & Bertrand, 2015).

In recent decades, antimicrobial resistance has emerged and evolved in many bacterial as the excessive use of antimicrobials in human and aquaculture systems (Kang et al., 2016; Li et al., 2013). The emergence of antimicrobial resistance in *Salmonella* to antibiotics such as ampicillin, chloramphenicol, and cotrimoxazole will further complicate the treatment and management of enteric fever (Ejaz et al., 2017). Strains that have been detected are resistant to some clinical first‐line antibiotics used in the treatment of severe *Salmonella* infections (Boonkhot, Tadee, & Patchanee, 2015). As antibiotic‐resistant bacteria can be directly transmitted through the food chain or transfer their antimicrobial resistance to human pathogens by mobile genetic elements, it is important to monitor antibiotic resistance among *Salmonella* isolates and control the risk.

Serotyping is a phenotypic characteristic that is a useful epidemiological marker for *Salmonella*. More than 2,600 serotypes have been reported (Abbott, Ni, & Janda, 2012). The prevalence of *Salmonella* serovars in different countries also varies over time and regions. For example, *Salmonella* Enteritidis is the most common serovar in the United States. Furthermore, some serovars are also more dangerous. For example, *S. enterica* serotype Typhimurium, which can cause various symptoms include diarrheal disease (Andrews & Ryan, 2015; El‐Tayeb, Ibrahim, Al‐Salamah, Almaary, & Elbadawi, 2017). Molecular subtyping of *Salmonella* is effective for epidemiological investigations of infections and outbreaks. Multilocus sequence typing (MLST) is a good method based on sequence analysis of some housekeeping genes. Given its high repeatability in globally dispersed laboratories, MLST is becoming an important method in the investigation of various pathogens including *Salmonella* (Yang et al., 2015). Researchers have found that many bacteria contain conserved repetitive intergenic consensus sequences, which have proven to be useful for subtyping pathogens. As a PCR‐based fingerprinting technique, ERIC‐PCR is easy, fast, and relatively cheap (Li, Liu, Li, Xu, & Zheng, 2017).

As eggs are a popular food and Guangdong is an economic center in south China with more than 110 million people, it is essential for us to understand the prevalence of *S. enterica* in this region. So far, little is known of the distribution of strains among eggs. The aim of our study was to determine the antimicrobial resistance, serotype, and genetic diversity of *S. enterica* in eggs from Guangdong, China. The information generated in this study will aid in evaluating the prevalence and population of *S. enterica* to ensure egg safety.

## 2 MATERIALS AND METHODS

### 2.1 Sample collection, culture of *Salmonella*

From June 2017 to June 2018, we collected 1,000 egg samples from farms and markets in Guangdong, China.

Isolation and identification of strains were carried out according to previous reports (Yang et al., 2015). Briefly, each egg was disinfected with 75% alcohol, the shell was carefully removed, and the egg yolks and whites were mixed. Then, 25 g of the sample was added to 225 ml of buffered peptone broth, and 1 ml of the solution was incubated in 10 ml of selenite cystine broth (SC) at 37°C and 10 ml of tetrathionate brilliant green broth (TTB) at 42°C for 24 hr. The SC and TTB cultures were streaked onto xylose–lysine–tergitol 4 (XLT4) selective agar plates and chromogenic *Salmonella* agar plates (37°C, 24 hr). Presumptive colonies were stabbed into a triple sugar iron slant and incubated at 37°C for 24 hr. Typical *Salmonella* phenotypes were further confirmed with API 20E test strips (BioMerieux French).

### 2.2 Antimicrobial susceptibility

As per the guidelines of the Clinical and Laboratory Standards Institute (CLSI, 2018), the susceptibility of the *Salmonella* isolates to antibiotic was examined by disk diffusion. Briefly, Mueller–Hinton agar and a panel of 14 antibiotic disks were selected for the resistance tests. These antibiotics are commonly used in agriculture and life. Them can cover among five classes: macrolides (azithromycin, AZM, 15 μg; erythromycin, ERY, 15 μg), aminoglycosides (gentamicin, GEN, 10 μg; kanamycin, KAN, 30 μg; streptomycin, SM, 10 μg), quinolones (ciprofloxacin, CIP, 5 μg; nalidixic acid, NA, 30 μg), β‐lactams (amoxicillin, AMC, 10 μg; ampicillin, AMP, 10 μg; cephazolin, CEP, 30 μg; penicillin, PEN, 10 μg; piperacillin, PIP, 10 μg), and tetracyclines (minocycline, MIN, 30 μg; tetracycline, TET, 30 μg). The results were expressed as sensitive (S), intermediate (I), and resistant (R), following the methodology of the CLSI.

### 2.3 Serotyping of *Salmonella* isolates

All the *Salmonella* isolates were serotyped by agglutination tests on the basis of somatic‐O and H antisera in accordance with the Kauffmanne–White scheme (Boonkhot et al., 2015).

### 2.4 Molecular subtyping

The strains were analyzed by MLST using seven housekeeping genes: *aroC*, *dnaN*, *hemD*, *hisD*, *purE*, *sucA*, and *thrA*. The PCR conditions were taken from the *Salmonella* MLST website and database (http://mlst.warwick.ac.uk/mlst/dbs/Senterica/; Jolley, Chan, & Maiden, 2004). Each reaction mixture included the following (total volume, 25 ml): 2 *PCR Mix (Qiagen), 12.5 μl; 1 μl each primer, dd H_2_O, 9.5 ml; and DNA template, 1 μl. The PCR conditions were as follows: 96°C for 5 min; 35 cycles for 96°C for 1 min; 55°C for 1 min; 72°C for 1 min; and final extension at 72°C for 10 min. The products were sequenced on BGI instrument. The seven gene sequences were uploaded to the MLST database for comparison to get allele numbers and define STs. The MLST evolution tree was built using the Kimura‐2‐parameter in Mega 6.0 (Tamura, Stecher, Peterson, Filipski, & Kumar, 2013).

We used the ERIC‐PCR universal primers (ERIC1: 5‐ATGTAAGCTCCTGGGGATTCAC‐3 and ERIC2: 5‐AAGTAAGTGACTGGGGTGAGCG‐3). The PCR reaction consisted of 12.5 μl 2* TaKaRa Taq Mix; 1 μM primers; and 100 ng template DNA. The PCR program was as follows: denaturation at 95°C, 5 min; 35 cycles of 94°C for 45 s, 52°C for 1 min, and 72°C for 3 min; and then 72°C for 10 min. The products were separated by electrophoresis (2.0% agarose gels) for 40 min at 90 V. Cluster result analysis used a numerical taxonomy and multivariate analysis software package, based on Dice's similarity coefficient (*SD*) (James Rohlf, 2000).

## 3 RESULTS

### 3.1 *Salmonella* in eggs

We isolated *Salmonella* from 54 (5.40%) out of 1,000 eggs. Of these, 38 strains were from farms and 16 were from markets. The strains were stored at −80°C in trypticase soy broth (20% glycerol). All positive samples and strains information are shown in Table S1.

### 3.2 Antibiotic susceptibilities

The *Salmonella* isolates were classified as sensitive, intermediate, or resistant based on the diameter of inhibition zones around antibiotic disks as specified by the CLSI. Ampicillin (AMP) resistance was the most common with 32 (59.26%) resistant strains. As shown in Table 1, our strains were resistant to β‐lactams antibiotic, including amoxicillin (37.04%), cephazolin (38.89%), penicillin (33.33%), and piperacillin (29.63%). Following this, similar levels of antibiotic resistances were shown by isolates to aminoglycosides and tetracyclines antibiotics, such as gentamicin (18.52%), kanamycin (53.70%), streptomycin (27.78%), minocycline (31.48%), and tetracycline (42.59%) were similar. Only a few strains were resistant to macrolides and quinolones antibiotics, for example azithromycin (9.26%), erythromycin (7.41%), ciprofloxacin (9.26%), and nalidixic acid (12.96%). Of note, only six isolates were L193 to any of the tested antibiotics, and most isolates were resistant to more than three antibiotics (Table S1). In particular, three strains were resistant to seven antibiotics (SalE4, SalE24, SalE54), and one was resistant to eight (SalE13).

**Table 1:** Antimicrobial resistance profiles of the *Salmonella* isolates

Antimicrobial agents	*Salmonella enterica* (*n* = 54)		
	No. (%) of R	No. (%) of I	No. (%) of S
Macrolides			
Azithromycin (AZM)	5 (9.26)	3 (5.56)	46 (85.18)
Erythromycin (ERY)	4 (7.41)	1 (1.85)	49 (90.74)
Aminoglycosides			
Gentamicin (GEN)	10 (18.52)	5 (9.26)	39 (72.22)
Kanamycin (KAN)	29 (53.70)	3 (5.56)	22 (40.74)
Streptomycin (SM)	15 (27.78)	2 (3.70)	37 (68.52)
Quinolones			
Ciprofloxacin (CIP)	5 (9.26)	2 (3.70)	47 (87.04)
Nalidixic acid (NA)	7 (12.96)	4 (7.41)	43 (79.63)
β‐lactams			
Amoxicillin (AMC)	20 (37.04)	2 (3.70)	32 (59.26)
Ampicillin (AMP)	32 (59.26)	3 (5.56)	19 (35.18)
Cephazolin (CEP)	21 (38.89)	4 (7.41)	29 (53.70)
Penicillin (PEN)	18 (33.33)	2 (3.70)	34 (62.97)
Piperacillin (PIP)	16 (29.63)	5 (9.26)	33 (61.11)
Tetracyclines			
Minocycline (MIN)	17 (31.48)	5 (9.26)	32 (59.26)
Tetracycline (TET)	23 (42.59)	1 (1.85)	30 (55.56)

Abbreviations: I, intermediate resistance; R, resistant; S, susceptibility.

### 3.3 Serotypes phenotype

**Figure 1:**
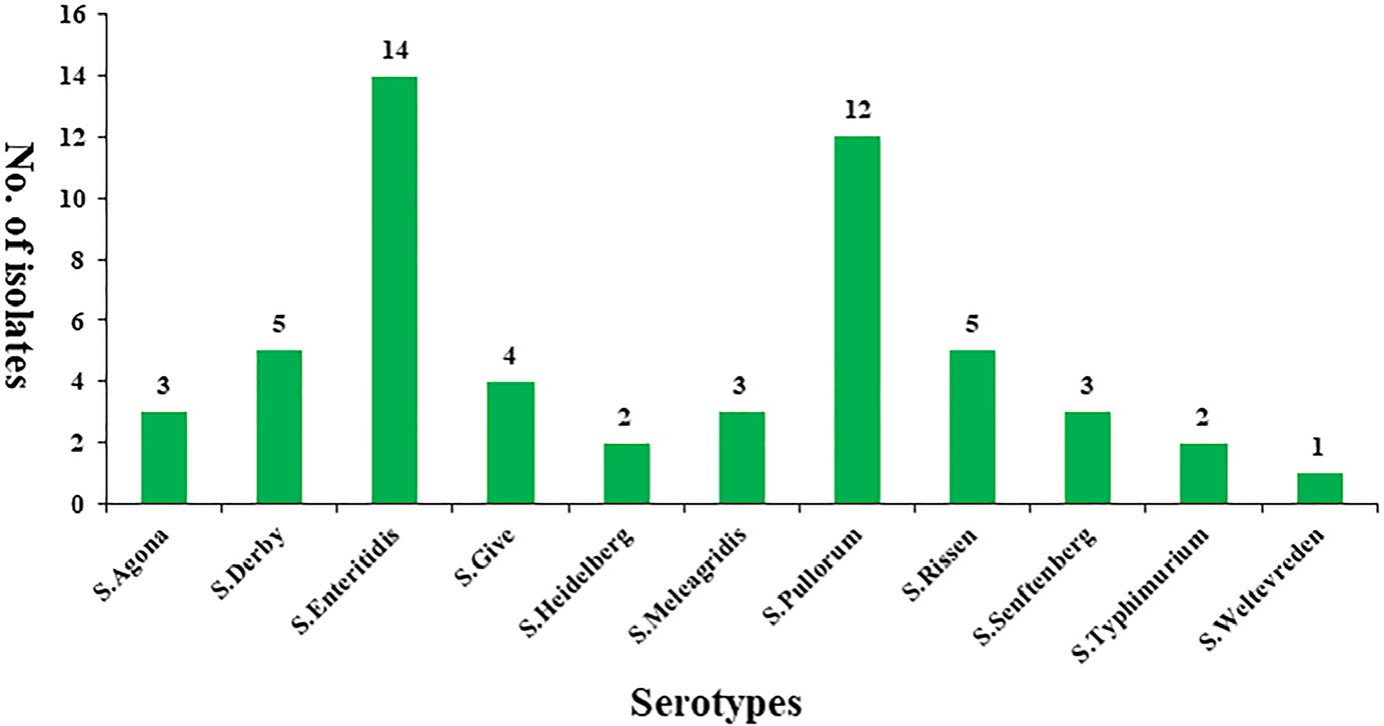
Distribution of *Salmonella* serotypes from egg samples

Somatic ‐O and ‐H antisera agglutination serotyped the 54 *Salmonella* isolates into 11 different serovars (Figure 1). Their distribution is as follows: *S.* Agona (3), *S.* Derby (5), *S.* Enteritidis (14), *S.* Give (4), *S.* Heidelberg (2), *S.* Meleagridis (3), *S.* Pullorum (12), *S.* Rissen (5), *S.* Senftenberg (3), *S.* Typhimurium(2), and *S.* Weltevreden (1).

### 3.4 Molecular diversity patterns

**Figure 2:**
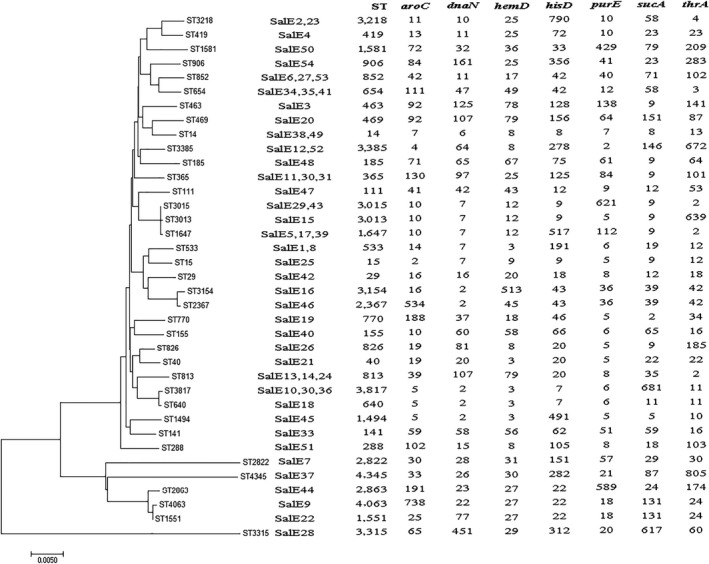
Multilocus sequence typing minimum evolution tree of the *Salmonella* isolates

After the seven housekeeping gene sequences were uploaded, the alleles were numbered and the isolates were assigned to 37 sequence types (STs) according to the *Salmonella* database. The numbers of each MLST locus were *aroC*: 29, *dnaN*: 27, *hemD*: 22, *hisD*: 28, *purE*: 24, *sucA*: 25, and *thrA*: 29. A minimum evolution tree was constructed using the concatenated sequences of each allele, as shown in Figure 2. The MLST results grouped the *Salmonella* isolates into three clusters. ST3315 (SalE28) formed its own cluster, which was widely separated from the others on the evolutionary tree.

**Figure 3:**
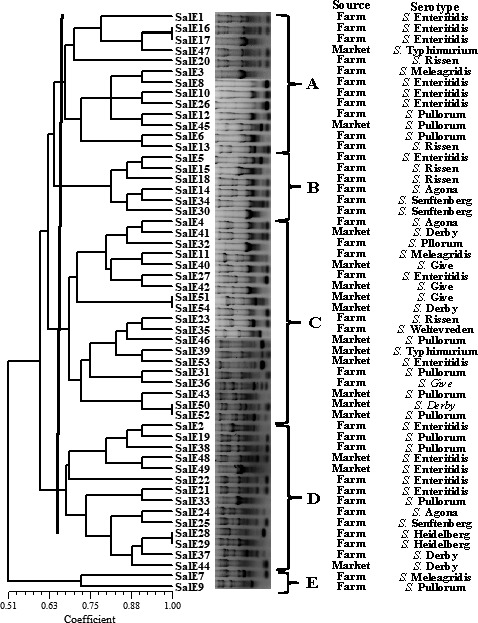
ERIC‐PCR DNA fingerprint analysis of the *Salmonella* isolates

The results of ERIC‐PCR analysis of the *Salmonella* isolates are shown in Figure 3. We found bands ranging in size from 100 bp to about 5,000 bp. The ERIC‐PCR patterns revealed that our isolates could be divided into five clusters (A, B, C, D, and E) with a relative similarity coefficient of 0.65. Analysis of the ERIC‐PCR found the isolates to be very diverse genetically. Meanwhile, the isolates belonging to clusters A and B were similar to the cluster in MLST typing, including SalE7, 9, 29, and 37. However, there was no evidence showing a relationship between source, serotype, and antibiotic resistance.

## 4 DISCUSSION

Eggs were an important host of *S. enterica*. In the United States alone, eggs were responsible for 80% of the 371 known *Salmonella* food poisoning cases from 1985 to 1999. In 2010, more than 2,000 people in the United States contracted food poisoning from consuming eggs contaminated with *Salmonella*. This led to the recall of more than 550 million eggs and resulted in heavy economic losses (Al, 2004). In China, about 70% of bacterial food poisoning is caused by *Salmonella*, with eggs and egg products accounting for 90% of these (Wu, Qin, Shi, & Zheng, 2004). Our data found that 7.6% and 3.2% of farm and market egg samples were contaminated with *Salmonella*, respectively. The market eggs may have a lower positive rate due to stricter access rules. The results were also much lower than contamination pork (41.8%) and other meats (Capuano, Mancusi, Capparelli, Esposito, & Proroga, 2013). For the egg samples, the contamination rate was similar to that seen in Iran and Shaanxi, China (Badouei, Ghalejooghi, & Madadgar, 2012; Wang et al., 2012). However, the data on the prevalence of *Salmonella* in eggs still show the risk of this organism infection and can be useful for control of egg consumption in China.

Resistance to different antibiotics has increasingly been reported in bacteria isolated from animals and humans and has become an important global issue. In particular, there is widespread dissemination of antimicrobial‐resistant strains among *S. enterica* (Hardjo Lugito & Cucunawangsih, 2017; Yang et al., 2015). Our susceptibility tests revealed the *Salmonella* isolates obtained from eggs were highly resistant to β‐lactams antibiotic, especially ampicillin. This resistance ratio coincides with many previous reports, finding that ampicillin resistance was most serious in *Salmonella* from some Asian countries (Chiou et al., 2014). Resistance to aminoglycosides and tetracyclines may be related to the use of feed containing large amounts of these antibiotics in chicken farms. Resistance to quinolones is also increasing along with prevalence of the resistance genes (Ben et al., 2017). Our isolates also showed resistance to other antibiotics including azithromycin (AZM) and erythromycin (ERY), which is a public health concern as these drugs are often used to treat human infections. In addition, many of the strains were multidrug resistant (MDR) with some even being resistant to more than seven antibiotics. Wang et al also showed that 84.6% of the *Salmonella* isolates from Shanghai, China, exhibited MDR and proved that resistance genes play an important role in MDR (Wang et al., 2017). Our findings indicate that it is necessary to continue monitoring the antimicrobial resistance of *Salmonella* isolates to help determine the appropriate antimicrobial therapy for patients infected with this pathogen.

*Salmonella enterica* has a wide variety of serotypes, and their distribution has strong regional variation. There are differences in the prevalence of *Salmonella* all over the world, but most common are *Salmonella* Enteritidis, *Salmonella* Typhimurium, and *Salmonella* Pullorum (Batista et al., 2015). For example, *S.* Senftenberg has been proved to be the dominant serovar in cooked meat products in Chinese Henan province (Yu, Jiang, Zhou, Wu, & Wu, 2014). The various serotypes have different virulence characteristics. Serotype Enteritidis is mainly transmitted through contaminated meat and egg products, which is the primary cause of human salmonellosis (Foley et al., 2011). This serotype was also the most widespread in our study (Figure 1). Serotype Typhi is defined as often being resistant to some first‐line recommended antibiotics (Hardjo Lugito & Cucunawangsih, 2017). There are 11 serovars found in our *Salmonella* isolates. These serovars have also been frequently isolated from different sources in China (Gong et al., 2014). *S.* Pullorum, which formed a large proportion of the isolates, are similar to strains isolated from gastroenteritis (Luo, Yi, Yao, Zhu, & Qin, 2017). As *Salmonella* serovars are closely related to pathogenicity, it is necessary to monitor changes in their distribution.

Molecular subtyping is widely used to analyze genetic diversity. MLST divided the *Salmonella* isolates into three clonal groups from different sources, which differed in all seven alleles profiled. We found that diverse STs mean that the isolates originated from more than one common ancestor and possess several distantly related STs. In the database, our STs were first isolated from diverse sources. For example, ST31, ST39, and ST40 were from pig farms, pig slaughterhouses, retail markets, and humans in China (Zheng et al., 2017). ST533, ST813, and ST640 were from clinics in Ethiopia and France. Our MLST results were used for analyzing correlations among the *Salmonella* strains. ERIC‐PCR fingerprinting has also been used to confirm epidemiological relationships between various *Salmonella* isolates (Li, Liu, et al., 2017). In Korea, researchers claimed that ERIC‐PCR combined with virulence profiling offered a rapid approach to characterize antimicrobial‐resistant *Salmonella* (Jin & Lee, 2017). We did not find a significant association between ERIC‐PCR clusters, source, and other characteristics. However, isolates in the last two MLST and ERIC‐PCR were similar. In general, MLST and ERIC‐PCR are useful as phylogenetic tools for investigations outbreaks of this pathogen.

## 5 CONCLUSION

*Salmonella enterica* food poisoning due to egg consumption is a longstanding problem. Our study is the first to comprehensively analyze the prevalence, antibiotic resistance, serotype, and molecular subtype of strains isolated from Guangdong eggs, which are an important local food of native. The results showed contamination ratio of *Salmonella* was 5.40%. Antibiotic resistance was widespread, especially for β‐lactam antibiotics. The isolates belonged to multiple serovars. MLST and ERIC‐PCR typing showed genetic similarity and diversity within those isolates. These results provide useful information to improve strategies to control and treat *Salmonella* infections.

## CONFLICT OF INTEREST

The authors declare that they do not have any conflict of interest.

## AUTHOR CONTRIBUTIONS

Tengfei Xie, Gang Wu are the common first authors, finished this article experiment and write the article together. Jing Zhao (Corresponding Author) give the idea and experiments support. Xujun He, Zengzhe Lai, Huatong Zhang help to finish the experiment on article.

## ETHICAL STATEMENT

Ethical Review: This study does not involve any human or animal testing.

## INFORMED CONSENT

Written informed consent was obtained from all study participants.
