# Supplementary material for: Antimicrobial resistance and genetic diversity of Salmonella enterica from eggs
Source: Food Sci Nutr. 2019 Aug 1;7(9):2847–53. doi: 10.1002/fsn3.1126 (PMC6766569; doi:10.1002/fsn3.1126)
Supplement: Supplementary file 1 [file FSN3-7-2847-s001.docx]

**Table S1. Results of soures, antimicrobial resistance, and serotyping of Salmonella isolates in this study**

|  | **Source** | **ST** | ***aroC*** | ***dnaN*** | ***hemD*** | ***hisD*** | ***purE*** | ***sucA*** | ***thrA*** | **Resistance profile** | **Serotype** |
| --- | --- | --- | --- | --- | --- | --- | --- | --- | --- | --- | --- |
| SalE1 | Farm | 533 | 14 | 7 | 3 | 191 | 6 | 19 | 12 | AZM-ERY-SM-AMC-PIP | *S.*Enteritidis |
| SalE2 | Farm | 3218 | 11 | 10 | 25 | 790 | 10 | 58 | 4 | GEN-KAN-SM-CEP | *S.*Enteritidis |
| SalE3 | Farm | 463 | 92 | 125 | 78 | 128 | 138 | 9 | 141 | KAN-AMP-CEP-PIP | *S.*Meleagridis |
| SalE4 | Farm | 419 | 13 | 11 | 25 | 72 | 10 | 23 | 23 | SM-NA-AMC-AMP-PEN-MIN-TET | *S.*Agona |
| SalE5 | Farm | 1647 | 10 | 7 | 12 | 517 | 112 | 9 | 2 | AMP-CEP | *S.*Enteritidis |
| SalE6 | Farm | 852 | 42 | 11 | 17 | 42 | 40 | 71 | 102 | AZM-SM-CEP-PIP-TET | *S.*Pullorum |
| SalE7 | Farm | 2822 | 30 | 28 | 31 | 151 | 57 | 29 | 30 | ERY-GEN-AMP-CEP | *S.*Meleagridis |
| SalE8 | Farm | 533 | 14 | 7 | 3 | 191 | 6 | 19 | 12 | KAN-SM-PEN-MIN-TET | *S.*Enteritidis |
| SalE9 | Farm | 4063 | 738 | 22 | 27 | 22 | 18 | 131 | 24 | GEN-SM-AMP-CEP | *S.*Pullorum |
| SalE10 | Farm | 3817 | 5 | 2 | 3 | 7 | 6 | 681 | 11 |  | *S.*Enteritidis |
| SalE11 | Farm | 365 | 130 | 97 | 25 | 125 | 84 | 9 | 101 | KAN-AMC-AMP-CEP-PEN-PIP | *S.*Meleagridis |
| SalE12 | Farm | 3385 | 4 | 64 | 8 | 278 | 2 | 146 | 672 | KAN-SM-NA-AMP-CEP | *S.*Pullorum |
| SalE13 | Farm | 813 | 39 | 107 | 79 | 20 | 8 | 35 | 2 | AZM-KAN-AMC-AMP | *S.*Rissen |
| SalE14 | Farm | 813 | 39 | 107 | 79 | 20 | 8 | 35 | 2 | SM-CIP-NA-AMC-AMP-CEP-PIP-MIN | *S.*Agona |
| SalE15 | Farm | 3013 | 10 | 7 | 12 | 9 | 5 | 9 | 639 | NA-AMP-PEN-TET | *S.Rissen* |
| SalE16 | Farm | 3154 | 16 | 2 | 513 | 43 | 36 | 39 | 42 |  | *S.*Enteritidis |
| SalE17 | Farm | 1647 | 10 | 7 | 12 | 517 | 112 | 9 | 2 | KAN-SM-CEP-PIP | *S.*Enteritidis |
| SalE18 | Farm | 640 | 5 | 2 | 3 | 7 | 6 | 11 | 11 | GEN-KAN-SM-AMP-MIN-TET | *S.*Rissen |
| SalE19 | Farm | 770 | 188 | 37 | 18 | 46 | 5 | 2 | 34 | AMC-AMP-CEP | *S.*Pullorum |
| SalE20 | Farm | 469 | 92 | 107 | 79 | 156 | 64 | 151 | 87 | KAN-AMC-AMP-PEN-PIP-TET | *S.*Rissen |
| SalE21 | Farm | 40 | 19 | 20 | 3 | 20 | 5 | 22 | 22 | GEN-KAN-AMC-AMP-MIN-TET | *S.*Enteritidis |
| SalE22 | Farm | 1551 | 25 | 77 | 27 | 22 | 18 | 131 | 24 | KAN-NA-PEN-PIP-TET | *S.*Enteritidis |
| SalE23 | Farm | 3218 | 11 | 10 | 25 | 790 | 10 | 58 | 4 | KAN-AMP-PEN | *S.*Rissen |
| SalE24 | Farm | 813 | 39 | 107 | 79 | 20 | 8 | 35 | 2 | AZM-ERY-KAN-AMP-PIP-MIN-TET | *S.*Agona |
| SalE25 | Farm | 15 | 2 | 7 | 9 | 9 | 5 | 9 | 12 | KAN-AMC-PEN | *S.*Senftenberg |
| SalE26 | Farm | 826 | 19 | 81 | 8 | 20 | 5 | 9 | 185 | KAN-NA-CEP | *S.*Enteritidis |
| SalE27 | Farm | 852 | 42 | 11 | 17 | 42 | 40 | 71 | 102 | AMC-AMP-CEP-PIP-MIN | *S.*Enteritidis |
| SalE28 | Farm | 3315 | 65 | 451 | 29 | 312 | 20 | 617 | 60 | AMP-PIP-MIN-TET | *S.*Heidelberg |
| SalE29 | Farm | 3315 | 65 | 451 | 29 | 312 | 20 | 617 | 60 | KAN-SM-AMC-CEP-PIP-TET | *S.*Heidelberg |
| SalE30 | Farm | 3817 | 5 | 2 | 3 | 7 | 6 | 681 | 11 | KAN-AMC-AMP-PEN | *S.*Senftenberg |
| SalE31 | Farm | 365 | 130 | 97 | 25 | 125 | 84 | 9 | 101 | SM-NA-AMP-PEN-MIN | *S.*Pullorum |
| SalE32 | Farm | 365 | 130 | 97 | 25 | 125 | 84 | 9 | 101 | AZM-KAN-CIP-AMC | *S.*Pullorum |
| SalE33 | Farm | 141 | 59 | 58 | 56 | 62 | 51 | 59 | 16 | GEN-KAN-AMC-PIP-MIN-TET | *S.*Pullorum |
| SalE34 | Farm | 654 | 111 | 47 | 49 | 42 | 12 | 58 | 3 | KAN-AMC-AMP-PEN-TET | *S.*Senftenberg |
| SalE35 | Farm | 654 | 111 | 47 | 49 | 42 | 12 | 58 | 3 |  | *S.*Weltevreden |
| SalE36 | Farm | 3817 | 5 | 2 | 3 | 7 | 6 | 681 | 11 | AMC-PEN | *S.*Give |
| SalE37 | Farm | 4345 | 33 | 26 | 30 | 282 | 21 | 87 | 805 | AMC-AMP-CEP-PIP-MIN-TET | *S.*Derby |
| SalE38 | Farm | 14 | 7 | 6 | 8 | 8 | 7 | 8 | 13 | TET | *S.*Pullorum |
| SalE39 | Market | 1647 | 10 | 7 | 12 | 517 | 112 | 9 | 2 | CIP-CEP-TET | *S.*Typhimurium |
| SalE40 | Market | 155 | 10 | 60 | 58 | 66 | 6 | 65 | 16 | KAN-AMP-PEN-TET | *S.*Give |
| SalE41 | Market | 654 | 111 | 47 | 49 | 42 | 12 | 58 | 3 | KAN-SM-AMP-CEP-PIP-MIN | *S.*Derby |
| SalE42 | Market | 29 | 16 | 16 | 20 | 18 | 8 | 12 | 18 |  | *S.*Give |
| SalE43 | Market | 3015 | 10 | 7 | 12 | 9 | 621 | 9 | 2 | KAN-AMC-AMP-TET | *S.*Pullorum |
| SalE44 | Market | 2863 | 191 | 23 | 27 | 22 | 589 | 24 | 174 | GEN-KAN-AMP-PEN-MIN-TET | *S.*Derby |
| SalE45 | Market | 1494 | 5 | 2 | 3 | 491 | 5 | 5 | 10 | KAN-AMP | *S.*Pullorum |
| SalE46 | Market | 2367 | 534 | 2 | 45 | 43 | 36 | 39 | 42 |  | *S.*Pullorum |
| SalE47 | Market | 111 | 41 | 42 | 43 | 12 | 9 | 12 | 53 | GEN-SM-AMC-AMP-PEN-MIN | *S.*Typhimurium |
| SalE48 | Market | 185 | 71 | 65 | 67 | 75 | 61 | 9 | 64 | GEN-KAN-AMP-CEP-PIP-TET | *S.*Enteritidis |
| SalE49 | Market | 14 | 7 | 6 | 8 | 8 | 7 | 8 | 13 |  | *S.* Enteritidis |
| SalE50 | Market | 1581 | 72 | 32 | 36 | 33 | 429 | 79 | 209 | KAN-SM-AMP-PEN-MIN-TET | *S.*Derby |
| SalE51 | Market | 288 | 102 | 15 | 8 | 105 | 8 | 18 | 103 | CEP-PEN | *S.*Give |
| SalE52 | Market | 3385 | 4 | 64 | 8 | 278 | 2 | 146 | 672 | ERY-CIP-AMC-PEN-MIN | *S.*Pullorum |
| SalE53 | Market | 852 | 42 | 11 | 17 | 42 | 40 | 71 | 102 | KAN-AMP-CEP-TET | *S.*Enteritidis |
| SalE54 | Market | 906 | 84 | 161 | 25 | 356 | 41 | 23 | 283 | GEN-KAN-CIP-AMP-CEP-MIN-TET | *S.*Derby |
